# Adenomatoid tumor of epididymidis: A case report

**DOI:** 10.1186/1757-1626-1-206

**Published:** 2008-10-02

**Authors:** Stylianos Kontos, Ioannis Fokitis, Antigoni Karakosta, Georgios Koritsiadis, Konstantinos Mitsios, Stamatios Koutsikos, Sotirios Koritsiadis

**Affiliations:** 1 Department of Urology, General Hospital of Nikea, 3D Mantouvalou Street, Nikea, 18454, Piraeus, Greece; 2 Department of Pathology, General Hospital of Nikea, 3D Mantouvalou Street, Nikea,18454, Piraeus, Greece; 3 First Department of Urology, Laiko General Hospital, University of Athens, School of Medicine, Athens, Greece

## Abstract

**Background:**

Adenomatoid tumors are regarded as distinctive benign mesothelial neoplasms of the paratesticular region, most commonly occuring at the tail of the epididymidis.

Because of its rarity, the clinical and histopathological aspects are discussed.

**Case presentation:**

We present the case of a 41-year-old patient with an adenomatoid tumour located in the tail of the left epididymis that referred to our department with gradual enlarged intrascrotal mass. The diagnosis was achieved by echography, and was confirmed by surgical excision and histological analysis.

**Conclusion:**

Due to its low incidence in intrascrotal pathology, we believe it is important for the physician to be aware of this interesting entity in order to make a differential diagnosis from other inflammatory processes and to adopt the proper surgical approach.

## Background

A variety of neoplasms derived from mesenchymal elements may arise on the patatesticular tissues. The diagnostic considerations may include carcinoma of rete testis, malignant mesothelioma, ovarian-type epithelial tumors, epididymidal carcinoma and metastatic carcinoma. Adenomatoid tumors were first described in 1945 by Golden et al as a small firm asymptomatic intrascrotal mass, with no pain or tenderness, occurring in third to fifth decades of life. The origin of the adenomatoid tumor is not clear, although the structural and immunohistochemical studies support the mesothelial origin. The histological pattern of the tumor is characterized by tubules, cords, and small nests, formed of cuboidal cells with vacuolated cytoplasm, and we may discriminate three kinds of adenomatoid tumor:plexiform, glandular and angiomatoid. The elected treatment is orchiectomy and epididymectomy, a probably aggressive surgical treatment which in combination with immunohistological confirmation are essential to resolve a formidable diagnostic challenge.

## Case presentation

A 41 years old man referred to our department for painless enlargement in the left hemiscrotum. Personal and familial history were unremarkable, without epididymitis, torsion or trauma. On physical examination, a hard, painless, firm, intrascrotal mass at the lower pole of the left testis was revealed, apparently distinct from the testis and arising directly from the surface of the left epididymis. Scrotal skin epididymis, spermatic cord, inguinal region and right testis were normal.

Ultrasonography confirmed a solid isoechoic lesion lying on the border between epididymal tail and lower pole of the left testis, without any disruption of the architecture of the testical parenchyma. The testicular parenchyma immediately adjacent to the mass showed slightly degreased echogenity compared with the parenchyma elsewhere. Preoperative laboratory investigation, including hemogram, blood chemistry studies were within normal limits. Plasma levels of *β*-hCG (*β*-subunit human chronic gonadotropin), *α*-fetoprotein (AFP), lactic dehydrogenase (LDH) were within normal ranges. The first clinical impression was a probable germ cell neoplasm.

The patient subsequently underwent a total left orchiectomy-epididymectomy, which revealed a white, crescent-shaped, well circumscribed tumor, about 1,5 cm, without involvement of the testicular parenchyma.

**Figure 1:**
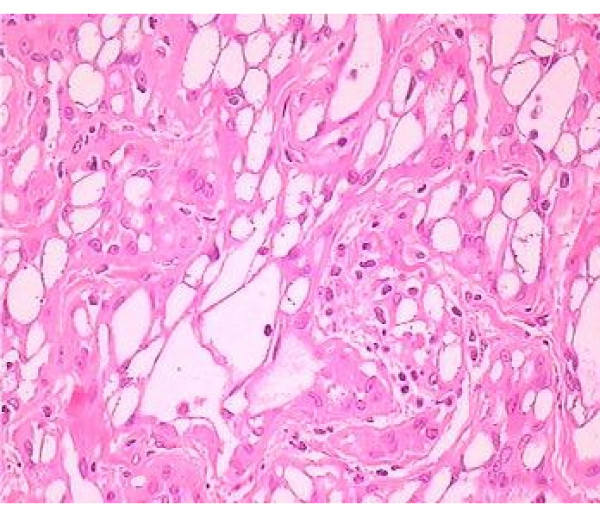
**Histological pattern of neoplasmatic cells**. Adenomatoid tumor with gaping spaces represented by cuboidal cells, with vacuolated cytoplasm (hematoxylene-eosine, ×40).

**Figure 2:**
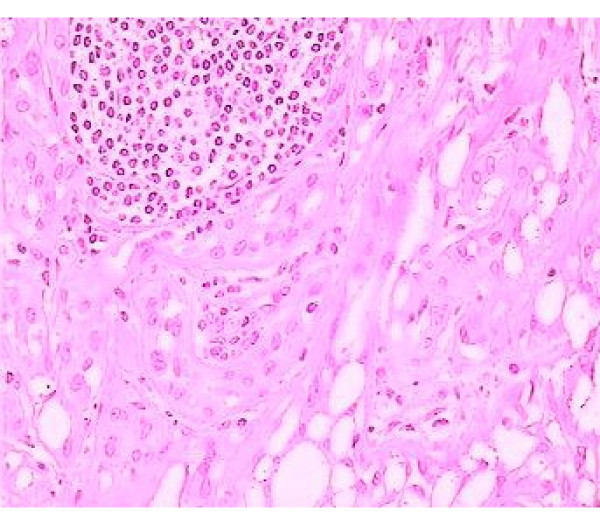
**Histological pattern of neoplasmatic cells**. Adenomatoid tumor with peripheral lemphoplasmatic infiltration particularly towards the periphery of the neoplasm, (hematoxylene-eosine, ×40).

Histological appearance of this paratesticular tumor is represented by cuboidal cells, with vacuolated cytoplasm and with gaping spaces (Fig. 1). The neoplasmatic cells had lymphoplasmacytic and eosinophilic infiltration, which varied in intensity, from scan to moderate dense, and especially the presence of prominent lymphoid aggregates, particularly towards the periphery of the neoplasm, is a helpful clue to the diagnosis of the adenomatoid tumor (Fig. 2). The cellular vacuoles had a signet ring-like appearance in some fields but epithelial type mucin was not present There was no mitotic activity and no suspicious neoplasmatic areas in testicular parenchyma was been detected.

**Figure 3:**
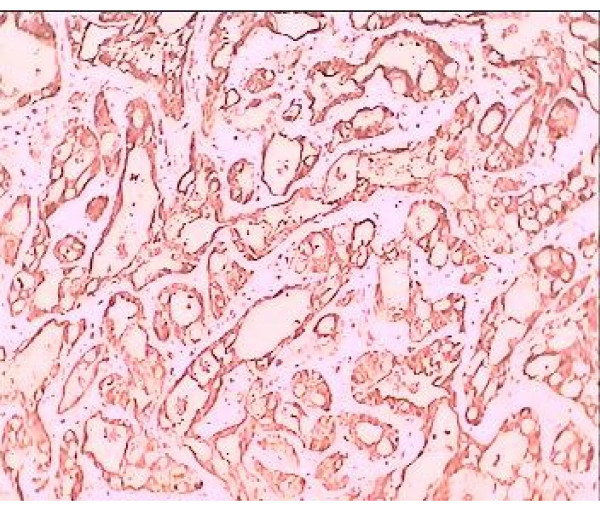
**Positive immunoreactivity to calretinin**. Adenomatoid tumor with cells performing positive immunoreactivity to calretinin-index of mesothelial origin (×40).

**Figure 4:**
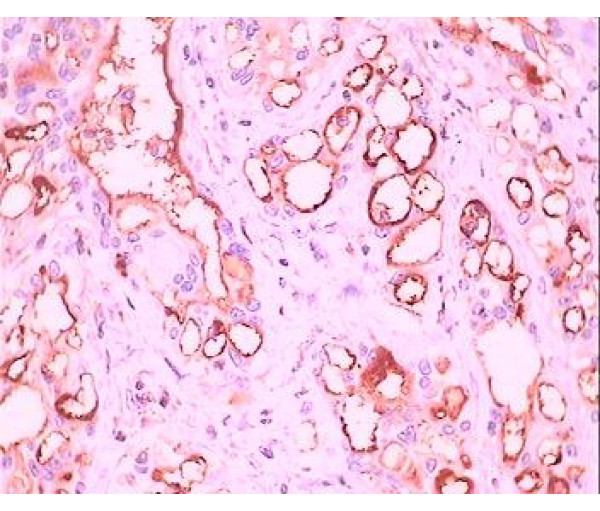
**Positive immunoreactivity to HMBE1**. Adenomatoid tumor with cells performing positive immunoreactivity to HMBE1-index of mesothelial origin (×40).

Immunohistochemical positivity for mesothelial marker HMBE1 (mouse anti-human antigen mesothelial cell) and calretinin confirm the mesothelial origin of the tumor (Fig. 3, 4). This tumor was diagnosed as an adenomatoid tumor in the epididymis and the patient underwent no additional treatment.

## Discussion

Paratesticular tumors are uncommon and comprise less than 5% of all intrascrotal tumors. Epididydimal epithelial tumors are a rare subtype of paratesticular tumors with the adenomatoid tumor being most common followed by the papillary cystadenoma and the leiomyoma [1]. These tumors usually arises in the epididymis, and approximately 14% of paratesticular adenomatoid tumors arise from the testicular tunica [2]. Except from epididymis, adenomatoid tumor can be located in spermatic cord, prostate, ejaculatory ducts, and scrotal capes and in female can be located in uterus, Fallopian tubes and ovarian area. It can be seen in all ages, but are most common in the third to forth decades of life [3].

Adenomatoid tumors present as a relatively small well demarked, without capsule nodule, less than 2 cm in diameter. The largest diameter that has been reported is 12 cm [2]. Most adenomatoid tumors of epididymis are asymptomatic and are found accidentally by the patient or by the physician on physical examination, as a non painful inscrotal mass commonly found in the tail of the epidydimis, which generally remains unchanged in size for years[4].

The tumor has a plethora of microscopic appearances, represented by three basic patterns:tubules, cords, and small nests, formed of cells that are cuboidal with vacuolated cytoplasm also characterized by peripheral eosinophilic and lymphatic infiltration [5]. Gaping spaces with no evident lining, representing a necrotic tubular component, and smaller spaces, representing ghost remnants of the typical vacuolar spaces, are major clues to the diagnosis. The differential diagnosis includes metastatic carcinoma, malignant mesothelioma, hystocytoid hemangioma, and carcinoma of the rete testis. Immunohistochemical confirmation with mesothelial-related markers (calretinin, HMBE1) is helpful in the differential with nonmeshothelial lesions [6].

The possible histogenesis of adenomatoid tumors has aroused controversy and our data, so far, are contradictory. However, the most recent investigations favor a mesothelial origin [7,8] and other pathologists have considered it to be a reaction to injury or inflammation. However, it is difficult to find such irritating factors in intrascrotal adenomatoid tumors. There is no case of malignant contact or presence of metastases [9].

In conclusion adenomatoid tumor of the epididymis is a distinctive clinical identity with much differential diagnosis, hard to distinguish; the cornerstone of accurate characterization of the tumor is still a comprehensive, traditional clinicopathologic approach, clinical history, routine light microscopy and immunohistochemical markers are crucial for the correct diagnosis.

## Abbreviations

*β*-hCG: *β*-human chorionic gonadotrophin; AFP: *α*-fetoprotein; LDH: lactic dehydrogenase; HMBE1: mouse anti-human antigen mesothelial cell

## Competing interests

The authors declare that they have no competing interests.

## Authors' contributions

All authors have made substantial contribution to concept this case report.

## Consent

Written informed consent was obtained from the patient for publication of this case report and accompanying images. A copy of the written consent is available for review by the Editor-in Chief of this journal.
